# Effect of erosive challenges on deciduous teeth undergoing restorative procedures with different adhesive protocols - an *in vitro* study

**DOI:** 10.1590/1678-7757-2017-0053

**Published:** 2018-01-16

**Authors:** Cristiane Meira Assunção, Marcelo Goulart, Tattiana Enrich Essvein, Nicole Marchioro dos Santos, Maria Carolina Guilherme Erhardt, Adrian Lussi, Jonas de Almeida Rodrigues

**Affiliations:** 1 Universidade Federal do Rio Grande do Sul, Faculdade de Odontologia, Divisão de Odontopediatria, Porto Alegre, RS, Brasil; 2 Universidade Federal do Rio Grande do Sul, Faculdade de Odontologia, Divisão de Dentística, Porto Alegre, RS, Brasil; 3 Universität Bern, Zahnmedizinische Kliniken, Klinik für Zahnerhaltung, Präventiv- und Kinderzahnmedizin, Bern, Switzerland

**Keywords:** Deciduous tooth, Erosive tooth wear, Adhesive, Tooth erosion, Tooth wear

## Abstract

**Objective:**

To evaluate the effect of erosive challenges on the tooth- restoration interface of deciduous teeth treated with different adhesive protocols.

**Material and Methods:**

Deciduous molars were cut mesiodistally, then embedded, abraded and polished (n=80). Samples were randomly divided according to the adhesive system used into: G1 (Adper Single Bond2^®^, etch-and-rinse), G2 (Universal Single Bond^®^, self-etching), G3 (OptibondFL^®^, etch-and-rinse with Fluoride) and G4 (BondForce^®^, self-etching with Fluoride). After standardized cavity preparation (2 mm diameter x 2 mm depth), adhesive systems were applied and samples were restored (composite resin Z350^®^). Half of the samples were exposed to erosive/abrasive cycles (n = 10, each adhesive group), and the other half (control group; n = 10) remained immersed in artificial saliva. For microleakage analysis, samples were submersed in methylene blue and analyzed at 40x magnifications. Cross-sectional microhardness (CSMH) was carried out (50 g/5 s) at 25 μm, 50 μm, and 100 μm from the eroded surface and at 25 μm, 75 μm, and 125 μm from the enamel bond interface.

**Results:**

Regarding microleakage, 7.5% of the samples showed no dye infiltration, 30% showed dye infiltration only at the enamel interface, and 62.5% showed dye infiltration through the dentin-enamel junction, with no difference between groups (p≥0.05). No significant difference was observed in CSMH at different depths (two-way ANOVA, p≥0.05).

**Conclusions:**

We did not observe significant changes in microleakage or CSMH after erosive/abrasive challenges in deciduous teeth treated with different adhesive protocols (etch-and-rinse and self-etching adhesives, with and without fluoride).

## Introduction

Erosive tooth wear (ETW) is a chemical-mechanical process that leads to the cumulative loss of hard dental tissue without the involvement of bacteria^4^. Enamel dissolution occurs both at the enamel/acid interface, as well as within a thin, softened, and partly demineralized layer of enamel, leading to mineral loss, and consequently to tooth substance loss^27^.

Tooth structure loss can cause tooth sensitivity, esthetics impairment, and loss of occlusal vertical dimension, leading to the indication of restorative treatment^29^. On the other hand, when exposing teeth with previous restorations to erosive and abrasive challenges, this can interfere in their durability^29^. Despite ETW being an emerging theme in recent studies, there are aspects that still need to be better explored, especially regarding the adhesive systems properties, restorative materials, and their application in deciduous teeth. The effect of erosive and abrasive challenges on enamel-restoration interfaces has not been deeply investigated up until now.

To obtain an adequate margin seal, it is necessary to apply adhesive systems under ideal conditions, thus ensuring the best restoration function without any breakdown between the tooth and the restoration^6^ ^,^ ^19^ ^,^ ^28^. Any failure at the bond interface can lead to microleakage, characterized by the infiltration of bacteria, fluids, chemical substances or ions between the tooth and the restorative material, as well as margin discoloration and even pulp inflammation^19^ ^,^ ^28^. Erosive tooth wear lesions in restored teeth are known by margin degradation and restorations rising above the level of the adjacent tooth surface.. This process starts at enamel and can develop until dentin exposure (rounding of cusps and grooves)^4^.

It is possible to assume the bonding success not only depends on adhesive proprieties, but on a combination of important aspects of the tooth substrate and the adhesive system^19^ ^,^ ^28^. Considering the enamel of deciduous teeth strongly reacts to acid etching, self-etching adhesive systems that have a higher pH and are less aggressive to the substrate, can be good for pediatric patients^31^. Besides these histological aspects, a systematic review, including *in vitro* studies that evaluated enamel and dentin bond strength, suggests that etch-and-rinse adhesives have a better performance in deciduous teeth compared to self-etch systems^15^.

Fluoride has been added to different dental materials to protect dental tissues. Some studies have investigated the effect of adhesive systems with fluoride on the inhibition of secondary caries, using pH cycling models to simulate demineralization and remineralization processes^13^ ^,^ ^14^ ^,^ ^22^ ^,^ ^23^. These studies showed the resistance of the tooth-restoration interface to acid increased when fluoride was present in the adhesive systems. A similar effect might be observed using erosive/abrasive cycles, but up until now, no study has tested this hypothesis in deciduous teeth.

Considering this knowledge gap, the hypothesis of this study was that the effect of erosive challenge on the enamel-restoration interface of deciduous teeth would be different from the selected adhesive protocols (etch-and-rinse and self-etching adhesives, with and without fluoride). The purpose was to evaluate the effect of erosive challenge on the enamel- restoration interface of deciduous teeth treated with different adhesive protocols using cross-sectional microhardness and microleakage.

## Material and methods

### Experimental design

The sample size measurement was based on Azevedo, et al.^3^ (2012), whose average difference percentage of CSMH loss was 26.5%. The statistical power was calculated at 80%, with 95% of confidence interval, resulting in 10 samples for each group (test and control, and 4 different adhesives). Consequently, 80 enamel samples from deciduous molars were selected for the experimental phase.

**Figure 1:**
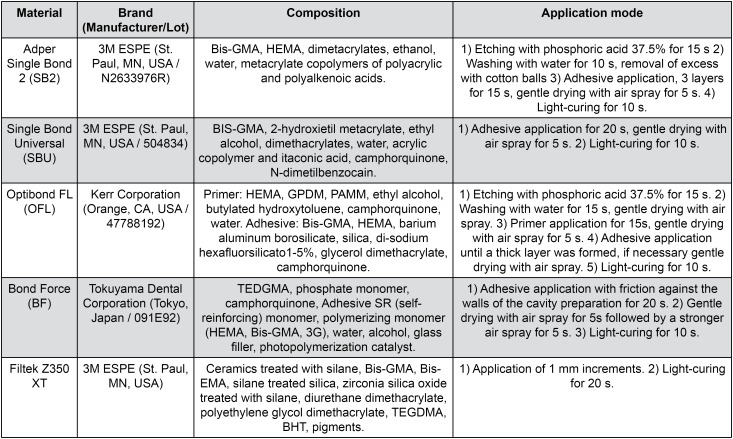
Description of the adhesive systems and the composite resin used in this study

The samples were randomly and equally divided according to the adhesive system used: G1 (etch- and-rinse, Adper Single Bond2^®^, 3M ESPE; St. Paul, MN, USA), G2 (self-etching, Universal Single Bond^®^, 3M ESPE; St. Paul, MN, USA), G3 (etch-and- rinse with Fluoride, OptibondFL^®^, Kerr Corporation; Orange, CA, USA) and G4 (self-etching with fluoride, BondForce^®^, Tokuyama Dental Corporation; Tokyo, Japan). Standard cavities were prepared, adhesive systems were applied and the samples were restored with composite resin (Filtek Z350 XT^®^, 3M ESPE; St. Paul, MN, USA) (Figure 1). Half of the samples were exposed to erosive and abrasive cycles (n = 10, each adhesive group), and the other half (control group, n = 10) remained immersed in artificial saliva^17^ during the experimental phase. In the experimental phase, the samples were stored in relative humidity at 4°C. At the end of the experimental phase, the group samples under test were exposed to 20 erosion cycles and 5 abrasion cycles. The tested variables were mineral loss (measured using CSMH) and marginal microleakage, which was measured by dye penetration degree.

### Sample preparation

In this study, sound deciduous molars were randomly selected from a group of extracted teeth stored at mineral solution (1.5 mmol/l CaCl_2_, 1.0 mmol/l KH_2_PO_4_, 50 mmol/l NaCl, pH = 7.0)^34^. The children's parents or legal guardians were informed on the use of the teeth for research purposes and their consent was obtained. The protocol was approved by the Research Ethics Committee of the Federal University of Rio Grande do Sul (Registration number 327.244).

The crowns were separated from the roots and cut mesiodistally, using a diamond disc at an Isomet^®^ Low Speed Saw (Buehler; Düsseldorf, Germany), so both lingual and buccal sides were used. The teeth fragments were embedded in polystyrene resin (Paladur^®^, Heraeus Kulzer GmbH; Hanau, Germany) inside PVC cylindrical molds. The samples were abraded using silicon carbide paper (grits of 1200, 2400, and 4000; Metadi - II^®^, Buehler Ltda; Lake Bluff, IL, USA) under constant irrigation with distilled water and polished with a diamond abrasive cloth (1¼ μm for 1 minute) under constant cooling (APL4^®^, Arotec Indústria e Comércio S/A; Cotia, SP, Brazil)^12^. This procedure removed approximately 200 pm of the enamel surface.

To select samples with the same mineral content, surface microhardness (KHN, 50 g/5 s, HMV-2T^®^, Shimadzu; Kyoto, Japan) was performed in the enamel surface, resulting in three indentations at 100 pm from each other^8^ ^,^ ^21^. The mean value of initial microhardness was KHN 332.79 (SD±1.89). Samples with KHN values different from the mean standard deviation values, scratches, fractures, exposed dentin or any other visible flaw, were excluded.

### Restorative procedures

Standardized cavity preparation was performed by perpendicularly introducing a cylindrical bur in the active area (diamond bur KG# 3131^®^, KG Sorensen Ind. e Com. Ltda; Barueri, SP, Brazil); when reaching enamel and dentin, the cavity depth was checked with a periodontal probe (2 mm diameter x 2 mm depth). Adhesive systems were applied and the samples were restored with composite resin, using the incremental technique (Figure 1). The light-curing was performed using an LED device (470 mW/cm^2^, Ortholux LED Curing Light^®^, 3M Unitek; Monrovia, CA, USA). Then, the samples were abraded (Sof-Lex, 3M ESPE; St. Paul, MN, USA) and polished (with felt discs and polish pastes, DiamondR FGM; Joinville, SC, Brazil). All samples were stored at 4°C under relative humidity until all measurements were performed^5^.

### Erosive and abrasive challenges

In the erosive challenge, the test group samples were immersed in 50 ml of Coca-Cola^®^ (pH 2.6, Coca- Cola Company; Curitiba, PR, Brazil) for 1 minute, at 25°C, under constant shaking, for four times a day, during five days. Between the cycles, the samples were washed with deionized water. The control group samples remained immersed in artificial saliva at room temperature (25°C).

All the samples of test groups were brushed using an electric toothbrush after the last cycle of the day (200 g force, for 1 minute), with a paste with fluoridated toothpaste (NaF, 1450 ppm, Colgate Total 12^®^, Colgate - Palmolive Comercial Ltda; São Bernardo do Campo, SP, Brazil) and artificial saliva (1:1)^7^ ^,^ ^16^ ^,^ ^20^ ^,^ ^32^ ^,^ ^33^.

### Microleakage analysis

For microleakage analysis, the area around the restoration was protected with nail varnish to only allow dye infiltration through the bonding margin. All samples were immersed in methylene blue 1% (pH 6.8) for 1 hour. Then, they were washed with deionized water and cross-sectioned (Isomet 1000^®^, Buehler Ltda; Lake Bluff, IL, USA). A parallel slice of each sample was obtained, containing half of the restoration.. Two trained and blinded examiners analyzed the samples using an optical microscope at 40x magnification. The qualitative microleakage analysis used the following scores: 0=no dye penetration, 1=dye penetration limited to the enamel, 2=dye penetration through the dentin-enamel junction^11^.

### Cross-sectional microhardness (CSMH)

**Figure 2:**
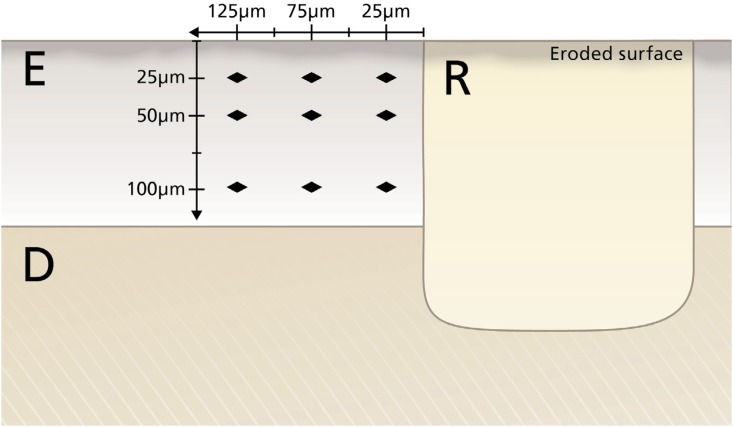
Schematic drawing of the cross-sectional surface microhardness (CSMH) measurements. E=enamel, D=dentin, R=restoration 2 mm diameter x 2 mm depth

After the microleakage evaluation, the samples were evaluated on microhardness. Cross-sectional microhardness (CSMH) was performed with nine indentations (KHN, 50 g/5 s, HMV-2T^®^, Shimadzu; Kyoto, Japan) in enamel located at 25 μm, 50 μm and 100 pm from the eroded surface and at 25 pm, 75 pm and 125 pm from the tooth-restoration interface (Figure 2)^13^.

### Statistical analysis

The normality of data distributions were evaluated using the Kolmogorof-Smirnov test. Considering data presented a non-normal distribution, non-parametric tests were used. Microleakage data were analyzed using the Kruskal-Wallis test and using CSMH between adhesive systems. The control and test groups were analyzed using two-way ANOVA non-parametric test. All the analyses were performed with the software program SPSS (Statistical Package for the Social Sciences, version 18).

## Results

Regarding microleakage, 7.5% of the samples showed no dye infiltration, 30% showed dye infiltration only at the enamel surface, and 62.5% showed dye infiltration with amelo-dentin junction. We observed no significant difference in microleakage in groups when using the Kruskal-Wallis test (p≥0.05; Table 1).

**Table 1:** Distribution frequency of microleakage scores in different adhesives systems, and in control and test groups (n=10; Kruskal- Wallis)

Score	G1	(SB)	G2	(SBU)	G3	(OFL)	G4	(BF)	Total	
	Test	Control	Test	Control	Test	Control	Test	Control		p value
	n(%)	n (%)	n (%)	n (%)	n (%)	n (%)	n (%)	n (%)	n(%)	
**0**	1(10)	1 (10)	1 (10)	1 (10)	1 (10)	1 (10)	0 (0)	0 (0)	**6 (7.5)**	0.255
**1**	2 (20)	4 (40)	2 (20)	4 (40)	5 (50)	2 (20)	3 (30)	2 (20)	**24 (30)**	
**2**	7 (70)	5 (50)	7 (70)	5 (50)	4 (40)	7 (70)	7 (70)	8 (80)	**50 (62.5)**	

We observed no significant differences in CSMH between the control and test groups at different depths, neither between different adhesive systems (two-way ANOVA, p≥0.05; Table 2).

**Table 2:** Mean values of cross-sectional surface microhardness (CSMH) measurements in different adhesive systems and control and tests groups, at each distance from the eroded surface (depth) and enamel bond margin (n=10; two-way ANOVA)

Distance from bond margin		G1 (SB2)		G2 (SBU)		G3 (OFL)		G4 (BF)			
	Depth	Test	Control	Test	Control	Test	Control	Test	Control	* *p* value	† *p* value
	25 μm	228.58 (60.27)	237.68 (47.22)	240.29 (76.38)	230.22 (100.63)	262.81 (38.62)	152.27 (94.28)	263.75 (46.99)	210.27 (40.15)	0.607	0.103
25μm	50 μm	249.89 (57.9)	272.7 (42.24)	247.09 (55.78)	255.89 (97.82)	278.1 (41.60)	159.91 (107.77)	275.49 (56.50)	252.52 (37.56)	0.960	0.634
	100 μm	280.26 (44.2)	275.49 (30.36)	278.24 (38.46)	245.64 (80.59)	277.58 (32.65)	158.35 (120.38)	278.72 (39.31)	280.12 (41.62)	0.419	0.112
	25 μm	259.7 (71.81)	253.02 (37.97)	263.54 (38.88)	247.52 (65.45)	253.99 (62.79)	155.47 (106.11)	250.06 (48.38)	253.85 (56.79)	0.947	0.797
75 μm	50 μm	289.94 (52.23)	258.37 (31.81)	238.7 (44.59)	268.81 (64.13)	286.95 (43.51)	157.91 (117.75)	264.38 (65.01)	268.27 (63.79)	0.533	0.581
	100 μm	264.85 (50.15)	271.92 (42.86)	266.68 (54.80)	281.34 (72.22)	277.46 (43.74)	162.60 (116.16)	280.47 (38.19)	271.93 (46.20)	0.852	0.613
	25 μm	259.75 (72.44)	263.25 (39.10)	271.69 (56.59)	230.55 (53.73)	265.3 (44.80)	155.72 (108.79)	240.21 (47.87)	230.06 (36.56)	0.321	0.166
125 μm	50 μm	279.75 (64.04)	289.99 (59.96)	261.68 (44.52)	266.77 (60.07)	278.75 (27.01)	163.25 (118.88)	269.78 (74.20)	251.69 (53.81)	0.735	0.478
	100 μm	243.22 (55.48)	268.16 (26.1)	243.21 (36.50)	269.15 (61.96)	279.63 (53.81)	153.77 (113.65)	270.27 (48.47)	263.36 (44.66)	0.387	0.574

Mean (DP)

* *p* value Comparsions among groups of adhesive protocols.

† *p* value Comparsions between test and control groups.

## Discussion

Erosive tooth wear (ETW), considered an emerging problem in oral health, have been increasingly prevalent among adults, adolescents and children^29^. Despite this fact, few studies have explored this subject in deciduous teeth, especially regarding the properties of restorative materials and their resistance to the erosive challenge. This study showed no significant differences in microleakage or CSMH after erosive/abrasive challenges in deciduous teeth treated with different adhesives.

Microleakage tests that have used organic and inorganic dyes to evaluate the tooth-restoration interface have been widely used because they are easy and quick to perform^2^. However, the results of these tests might have been influenced by variations in methodology (dye type and concentration, immersion duration, method of analysis, cavity preparation dimension). Therefore, these variables could make result comparison more difficult, which might lead to uncertain and incorrect conclusions^2^ ^,^ ^10^ ^,^ ^25^ ^,^ ^31^. Despite the existence of such variations, microleakage tests using dyes seem to evaluate the differences between materials in laboratory studies adequately, thus providing an improved basis for clinical trials. Choosing methylene blue 1% (pH 6.8) ensured that no other acidic exposure would interfere in the outcome of this study^10^.

In our study, significant dye penetration was found in all groups. Some studies have tested the same adhesive systems and have also observed a high degree of dye penetration^2^ ^,^ ^30^. Other authors have not observed statistically significant differences in microleakage when comparing different adhesives^1^ ^,^ ^2^ ^,^ ^24^ ^,^ ^30^.

Some experimental models with longer immersion in dyes or a long-term evaluation of these restorations could better compare the microleakage of the tooth- restoration interface in different adhesive systems. In our study, we exposed the samples to 20 erosion cycles and 5 abrasion cycles, leading to initial erosive tooth wear, which was not significantly different in the tested adhesive systems.

Considering the different adhesives protocols, a recent systematic review of *in vitro* studies evaluated bond strength in deciduous teeth. The statistical analysis of the grouped immediate bond strength data showed that etch-and-rinse adhesives bonded better to sound enamel and dentin substrates than self-etch systems^15^. It described a wide range of sample sizes and adhesive protocols, so studies with less bias should be considered by professionals when deciding for one of the many adhesives options. The fluoride addition in adhesive systems showed protective effects on the enamel-restoration interface, considering pH-cycling models^13^ ^,^ ^14^ ^,^ ^22^ ^,^ ^23^. Guedes, et al.^9^ (2016) evaluated the effect of erosive pH cycling with solutions that simulate dental erosion on Martens hardness of bovine dentin restored with fluoride-releasing adhesive systems. This study concluded that fluoride from self-etching adhesive systems One Up Bond F^®^ (Tokuyama Dental Corporation; Tokyo, Japan) and Clearfil SE Protect^®^ (Kuraray America, Inc.; New York, NY, USA) could have some positive effect on erosive lesions early- stages^9^. Sato, et al.^26^ (2016) evaluated the acid-base resistant zone at the adhesive/enamel interface of self-etching adhesives with or without prior phosphoric acid etching^26^. They restored samples of third molars and pre molars by carrying out different self-etching adhesives protocols and pH cycling. The authors concluded that enamel beneath the bonding interface was more susceptible to acid dissolution in Scotchbond Universal^®^ adhesive (3M ESPE; St. Paul, MN, USA) and Clearfil BOND SE ONE^®^ (Kuraray America, Inc.; New York, NY, USA). In the case of the self-etching adhesives and universal adhesives, enamel etching is useful to improve the interfacial quality^26^. This study evaluated deciduous enamel bonding margin after erosive/abrasive challenges, and no significant difference was demonstrated in etch-and-rinse or self-etching adhesives, with or without fluoride on composition.

With the erosive/abrasive challenge used in this study, we observed no statistically significant differences regarding microleakage or CSMH in the different adhesive systems used (with and without fluoride, etch-and-rinse, and self-etching). Microhardness evaluations, either superficial or cross-sectional, imply quantitative measures that can evaluate minimum changes on mineral content; it is a widely used method to compare different treatments in erosive/abrasive protocols. By using Coca-Cola^®^ (pH 2.6, Coca-Cola Company; Curitiba, PR, Brazil) and following the previously described protocol (1 minute at 25°C under constant shaking), we aimed at getting closer to *in vivo* conditions, simulating the children's acid beverage intake. A study with bovine teeth using an erosion model showed some significant differences in microhardness values, especially in sample restoration with fluoride releasing material, such as glass ionomer^35^. A pH cycling study that simulated caries found significant differences between adhesive systems with and without fluoride. The microhardness values of dentin at 50 μm were similar between one self-etching adhesive system with fluoride and a conventional glass ionomer cement^14^. On the other hand, the same authors investigated different restorative techniques exposed to a cariogenic challenge in an *in situ* study, and have not found differences between adhesive systems with or without fluoride, and the group restored with conventional glass ionomer cement showed higher CSMH values^13^.

Several studies comparing toothpastes with and without fluoride in erosion-abrasion models showed that fluoride formulations applied on enamel had a protective effect on teeth. It was possible to observe lower surface loss on samples brushed with fluoride toothpaste compared to samples with no fluoride toothpaste^17^ ^,^ ^18^. The main effect of fluoride on erosion/ abrasion cycles is the increase in enamel resistance to future acid exposure, as there is no remineralization of the softened layer. The fluoride's protective effect was present both in test and control groups of this study by applying a paste containing NaF fluoride toothpaste during abrasions cycles.

The fact that we have not observed significant difference in CSMH is due to the removal of the softened layer by the five abrasion cycles and the short-term evaluation after 20 erosion cycles. We could consider such characteristic as one of the limitations of this study. The amount of fluoride released from adhesives with fluoride is not usually known and may not be high enough to reduce demineralization in erosive challenges. In this study, the fluoride content of the adhesive systems was not enough to have a protective effect on the enamel-restoration interface. The short term evaluation could be another limitation of the study. It could be expected that, after a longterm evaluation with more erosion/abrasion cycles and measurements of nanohardness closer than 25 μm from the enamel bond margin, some differences could be observed among the adhesive systems tested in this study. The evaluation of surface loss with profilometry analysis could provide additional information on the effect of erosive tooth wear on deciduous teeth restored with different adhesive systems.

The authors state that erosive tooth wear (ETW) is a condition of growing importance even in primary dentition, requiring preventive to restorative interventions. The selection of the most adequate adhesive system to restore deciduous teeth exposed to ETW is an important step in ensuring the success of restorative treatments.

## Conclusion

Therefore, based on the results of this *in vitro* study, the addition of fluoride to adhesive systems did not interfere in the investigated outcomes (microleakage and CSMH). The different adhesives protocols (etch- and-rinse or self-etching) did not show any difference on enamel bonding interface evaluation after erosive/abrasive challenges.

## References

[1] Amaral CM, Hara AT, Pimenta LA, Rodrigues AL (2011). Microleakage of hydrophilic adhesive systems in class V composite restorations. Am J Dent..

[2] Amarante de Camargo DA, Sinhoreti MA, Correr-Sobrinho L, Sousa MD, Consani S (2006). Influence of the methodology and evaluation criteria on determining microleakage in dentin-restorative interfaces. Clin Oral Investig..

[3] Azevedo DT, Faraoni-Romano JJ, Derceli JR, Palma-Dibb RG (2012). Effect of Nd:YAG laser combined with fluoride on the prevention of primary tooth enamel demineralization. Braz Dent J..

[4] Carvalho TS, Colon P, Ganss C, Huysmans MC, Lussi A, Schlueter N (2015). Consensus report of the European Federation of Conservative Dentistry: erosive tooth wear - diagnosis and management. Clin Oral Investig..

[5] Cheaib Z, Lussi A (2011). Impact of acquired enamel pellicle modification on initial dental erosion. Caries Res..

[6] Correr GM, Puppin-Rontani RM, Correr-Sobrinho L, Sinhoret MA, Consani S (2004). Effect of sodium hypochlorite on dentin bonding in primary teeth. J Adhes Dent..

[7] Cruz JB, Lenzi TL, Tedesco TK, Guglielmi CA, Raggio DP (2012). Eroded dentin does not jeopardize the bond strength of adhesive restorative materials. Braz Oral Res..

[8] Cury JA, Rebelo MA, Del Bel Cury AA, Derbyshire MT, Tabchoury CP (2000). Biochemical composition and cariogenicity of dental plaque formed in the presence of sucrose or glucose and fructose. Caries Res..

[9] Guedes AP, Moda MD, Suzuki TY, Godas AG, Sundfeld RH, Briso AL (2016). Effect of fluoride-releasing adhesive systems on the mechanical properties of eroded dentin. Braz Dent J..

[10] Heintze SD (2013). Clinical relevance of tests on bond strength, microleakage and marginal adaptation. Dent Mater..

[11] International Standardization Organization (1994). ISO/TR 11405:1994: Dental materials - guidance on testing of adhesion to tooth structure.

[12] Johansson AK, Sorvari R, Birkhed D, Meurman JH (2001). Dental erosion in deciduous teeth - an *in vivo* and *in vitro* study. J Dent..

[13] Kirsten GA, Rached RN, Mazur RF, Vieira S, Souza EM (2013). Effect of open-sandwich vs. adhesive restorative techniques on enamel and dentine demineralization: an in situ study. J Dent..

[14] Kirsten GA, Takahashi MK, Rached RN, Giannini M, Souza EM (2010). Microhardness of dentin underneath fluoride-releasing adhesive systems subjected to cariogenic challenge and fluoride therapy. J Dent..

[15] Lenzi TL, Gimenez T, Tedesco TK, Mendes FM, Rocha RO, Raggio DP (2016). Adhesive systems for restoring primary teeth: a systematic review and meta-analysis of *in vitro* studies. Int J Paediatr Dent..

[16] Levy FM, Magalhães AC, Gomes MF, Comar LP, Rios D, Buzalaf MA (2012). The erosion and abrasion inhibiting effect of TiF(4) and NaF varnishes and solutions on enamel *in vitro*. Int J Paediatr Dent..

[17] Lussi A (2009). Dental erosion - novel remineralizing agents in prevention or repair. Adv Dent Res..

[18] Magalhães AC, Rios D, Delbem AC, Buzalaf MA, Machado MA (2007). Influence of fluoride dentifrice on brushing abrasion of eroded human enamel: an *in situ/ex vivo* study. Caries Res..

[19] McDonough WG, Antonucci JM, He J, Shimada Y, Chiang MY, Schumacher GE (2002). A microshear test to measure bond strengths of dentin-polymer interfaces. Biomaterials..

[20] Moretto MJ, Magalhães AC, Sassaki KT, Delbem AC, Martinhon CC (2010). Effect of different fluoride concentrations of experimental dentifrices on enamel erosion and abrasion. Caries Res..

[21] Paes Leme AF, Tabchoury CP, Zero DT, Cury JA (2003). Effect of fluoridated dentifrice and acidulated phosphate fluoride application on early artificial carious lesions. Am J Dent..

[22] Pedrosa VO, Flório FM, Turssi CP, Amaral FL, Basting RT, França FM (2012). Influence of pH cycling on the microtensile bond strength of self-etching adhesives containing MDPB and fluoride to dentin and microhardness of enamel and dentin adjacent to restorations. J Adhes Dent..

[23] Peris AR, Mitsui FH, Lobo MM, Bedran-Russo AK, Marchi GM (2007). Adhesive systems and secondary caries formation: assessment of dentin bond strength, caries lesions depth and fluoride release. Dent Mater..

[24] Pilo R, Ben-Amar A (1999). Comparasion of microleakage for three one-bottle and three multiple-step dentin bonding agents. J Prosthet Dent..

[25] Raskin A, D'Hoore W, Gonthier S, Degrange M, Déjou J (2001). Reliability of *in vitro* microleakage tests: a literature review. J Adhes Dent..

[26] Sato T, Takagaki T, Matsui N, Hamba H, Sadr A, Nikaido T (2016). Morphological evaluation of the adhesive/enamel interfaces of two-step self-etching adhesives and multimode one-bottle self-etching adhesives. J Adhes Dent..

[27] Shellis RP, Barbour ME, Jesani A, Lussi A (2013). Effects of buffering properties and undissociated acid concentration on dissolution of dental enamel in relation to pH and acid type. Caries Res..

[28] Silva Telles PD, Aparecida M, Machado M, Nör JE (2001). SEM study of a self-etching primer adhesive system used for dentin bonding in primary and permanent teeth. Pediatr Dent..

[29] Taji S, Seow WK (2010). A literature review of dental erosion in children. Aust Dent J..

[30] Toledano M, Cabello I, Yamauti M, Giannini M, Aguilera FS, Osorio E (2012). Resistance to degradation of resin-dentin bonds produced by one-step self-etch adhesives. Microsc Microanal..

[31] Van Meerbeek B, Peumans M, Poitevin A, Mine A, Van Ende A, Neves A (2010). Relationship between bond-strength tests and clinical outcomes. Dental Materials.

[32] Voronets J, Lussi A (2010). Thickness of softened human enamel removed by toothbrush abrasion: an *in vitro* study. Clin Oral Investig..

[33] Wang L, Casas-Apayco LC, Hipólito AC, Dreibi VM, Giacomini MC, Bim O (2014). Effect of simulated intraoral erosion and/or abrasion effects on etch-and-rinse bonding to enamel. Am J Dent..

[34] Zero DT, Rahbek I, Fu J, Proskin HM, Featherstone JD (1990). Comparison of the iodide permeability test, the surface microhardness test, and mineral dissolution of bovine enamel following acid challenge. Caries Res..

[35] Zhou SL, Zhou J, Watanabe S, Watanabe K, Wen LY, Xuan K (2012). *In vitro* study of the effects of fluoride-releasing dental materials on remineralization in an enamel erosion model. J Dent..

